# Visually detectable pointer deviation of the nextar surgical navigation system with c-arm, o-arm and ct imaging: a bench-top study using a two-dimensional phantom

**DOI:** 10.1186/s12891-026-10696-1

**Published:** 2026-11-02

**Authors:** Richard Zaccaria, Eduardo Suero, Christopher Lampert, Dominic Simon, Rouven Neudeck, Stefan Hauck, Annabelle Weigert, Paul Reidler, Wolfgang Böcker, Bernd Wegener

**Affiliations:** 1 Musculoskeletal University Center Munich (MUM), LMU University Hospital, LMU Munich, Marchioninistr. 15, 81377 Munich, Germany; 2 BG Unfallklinik Murnau, Professor-Küntscher-Straße 8, 82418 Murnau am Staffelsee, Germany; 3 Department of Radiology, LMU University Hospital, LMU Munich, Marchioninistr. 15, 81377 Munich, Germany

**Keywords:** Surgical navigation, Target registration error, Imaging modality, C-arm, O-arm, Computed tomography, Bench-top study, Phantom

## Abstract

**Background:**

The accuracy of surgical navigation systems is critical for their safe and effective clinical use. This bench-top study evaluated whether a positional deviation of the pointer tip from a 1-mm target could be detected on the display of the NextAR surgical navigation system (Medacta International SA, Switzerland) when image-to-patient registration was based on C-arm, O-arm or CT imaging, and whether such a detectable deviation depended on the distance between the fiducial reference marker and the target. The endpoint was a visual observation of the pointer on the display and not an independently measured target registration error (TRE).

**Methods:**

A flat two-dimensional phantom (millimetre graph paper on a wooden board) carried two parallel rows of 20 steel nails with 1-mm heads. Adjacent nails were 10 mm apart and the rows 120 mm apart. The fiducial was fixed midway between the rows at the start of the grid and defined the origin. The phantom was imaged with a Ziehm Vision RFD 3D C-arm, a Medtronic O-arm MVS O2 and a Siemens SOMATOM Drive CT. Ten image acquisitions, each followed by a new image-to-patient registration, were performed per modality. For each nail within the imaged volume, the tip of a navigated pointer was placed on the nail head and the operator visually inspected the NextAR three-dimensional display for a deviation. NextAR provides no numerical deviation value. No artificial registration error was introduced, and the pointer tip was not independently measured. The primary endpoint, visually detectable pointer deviation (hereafter detectable deviation), was a visible offset of the pointer tip from the centre of the nail head on the display. The threshold of about 1 mm is an approximate limit given by the size of the nail head and the millimetre grid and was neither calibrated nor independently measured. Results are descriptive, with exact two-sided Clopper–Pearson 95% confidence intervals.

**Results:**

No detectable deviation was observed in any acquisition (0/10 per modality; exact 95% CI 0–30.9%; overall 0/30, 95% CI 0–11.6%). The maximum fiducial-to-target distances evaluated were 143 mm with C-arm, 152 mm with O-arm and 199 mm with CT imaging. The intervals refer to acquisitions under fixed bench-top conditions.

**Conclusions:**

Under the controlled conditions of this two-dimensional bench-top experiment, no deviation was visible on the NextAR display with any of the three imaging modalities. Because the endpoint was a visual assessment limited to about 1 mm and was not independently measured against an external reference, the study does not establish submillimetre accuracy, equivalence between modalities, absence of distance-related effects, or clinical reliability. Verification in anatomically appropriate cadaveric and clinical settings is required.

**Clinical trial number:**

Not applicable.

## Background

Surgical navigation systems are increasingly integrated into clinical practice with the aim of improving surgical accuracy and patient safety [1]. Their use is particularly established in spinal surgery, where navigation-assisted techniques may improve pedicle screw placement accuracy and reduce the risk of malposition and associated neurological or vascular complications [2, 3]. The accuracy of pedicle screw placement in thoracolumbar fractures has been assessed with postoperative CT [4].

Image-guided surgical navigation systems provide spatial orientation based on medical imaging data [1, 5]. To achieve this, image datasets acquired by CT or intraoperative fluoroscopic imaging must be registered to the patient anatomy. A typical system combines a patient-fixed reference marker, an optical tracking system for instrument localization, and navigation software that calculates and displays the spatial relationship between surgical instruments and the registered anatomy [6].

In clinical practice, preoperative CT and intraoperative imaging modalities such as C-arm and O-arm systems are used for navigation [7–9]. These modalities differ in image acquisition and reconstruction characteristics, which may affect the quality of navigation data [7, 10]. Direct comparisons of navigation accuracy between imaging modalities remain limited [10, 11]. Previous studies have reported that navigation accuracy may vary according to imaging modality and other technical or clinical factors [7, 10–13], and the modalities also differ in radiation exposure [14].

The literature further indicates that target registration error (TRE) may increase with increasing distance between a target and the reference marker [15], and that navigation accuracy and precision also depend on the camera distance and the number of active markers [16]. The present study therefore evaluated whether a positional deviation of the pointer tip from a 1-mm target could be visually detected on the NextAR display (termed “visually detectable pointer deviation”) when C-arm, O-arm and CT datasets were used for image-to-patient registration, and whether this depended on the fiducial-to-target distance. A controlled two-dimensional phantom was used.

The study was deliberately designed as a detection study rather than as an independent continuous TRE validation study. No artificial registration error was introduced, and no external measurement system was used to establish a continuous ground-truth TRE. The endpoint of this study is therefore a visually observed pointer deviation and not a measured TRE.

## Methods

### Study design

This experimental bench-top study evaluated the visually detectable pointer deviation of the NextAR optical surgical navigation system (Medacta International SA, Castel San Pietro, Switzerland) after image-to-patient registration using three imaging modalities: an intraoperative C-arm (Ziehm Vision RFD 3D, Ziehm Imaging, Nuremberg, Germany) [17], an intraoperative O-arm (O-arm MVS O2, Medtronic, Minneapolis, USA) [18] and a CT scanner (SOMATOM Drive, Siemens Healthineers, Erlangen, Germany) [19]. Ten image acquisitions were performed for each modality. Each acquisition included a new image-to-patient registration; the phantom and the fiducial were not repositioned between acquisitions.

### Phantom and measurement geometry

The phantom consisted of a DIN A4 sheet (210 × 297 mm) of millimetre graph paper fixed with the targets to a flat wooden board. The targets were steel nails with a measured head diameter of 1 mm, driven through the paper into the board. Two parallel rows of 20 nails each were placed. Adjacent nails within a row were 10 mm apart, and the two rows were 120 mm apart. Nail positions were set by reading the millimetre grid; the placement tolerance of the nails and the dimensional accuracy of the printed grid were not independently verified.

**Fig. 1:**
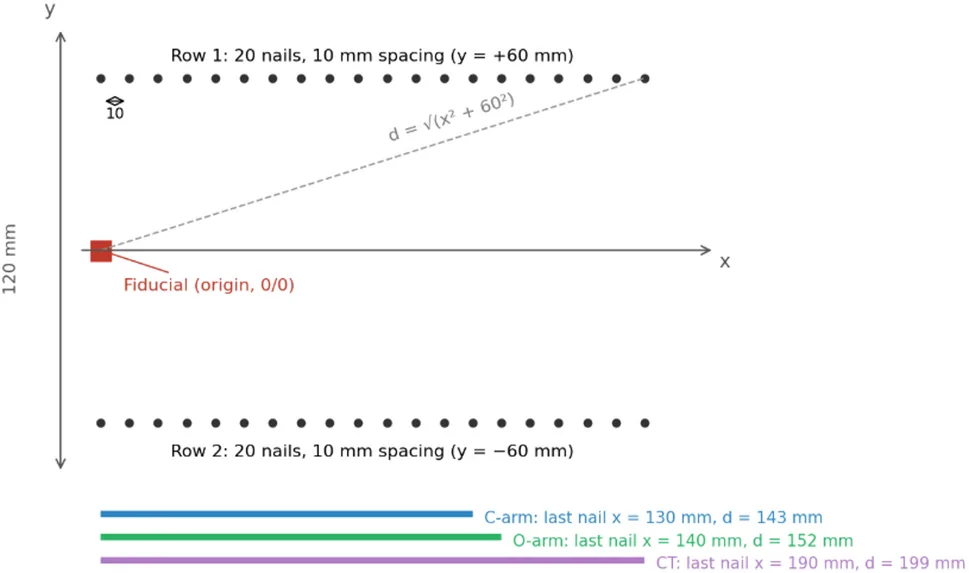
Dimensioned schematic of the phantom (top view, not to scale for the board). Two rows of 20 steel nails (head diameter 1 mm) with 10-mm spacing and 120 mm between the rows. The fiducial sits midway between the rows at the start of the grid and defines the origin. The coloured bars show the extent of the nails within the imaged volume for each modality and the resulting maximum fiducial-to-target distance d

The fiducial (patient reference marker) was rigidly fixed at the start of the grid, exactly midway between the two rows (60 mm from each row), and served as the origin of the coordinate system (0/0). The x-axis ran along the nail rows and the y-axis across them. The nails were located at x = 0, 10, …, 190 mm and y = + 60 mm and − 60 mm. The in-plane fiducial-to-target distance was calculated from the grid coordinates as d = √(x² + y²); it ranged from 60 mm (x = 0) to 199 mm (x = 190 mm). The phantom and the fiducial remained in the same position throughout the measurement series and the phantom was fixed to the board during all acquisitions. A dimensioned schematic is shown in Fig. 1. Because the phantom was flat, only in-plane positional deviations could be assessed. Depth, angular and trajectory errors relevant to procedures such as pedicle screw placement could not be assessed.

### Imaging modalities and acquisition

The phantom was imaged sequentially with the three modalities using the standard 3D acquisition settings of each system. The retained imaging records were dated 28 May 2025 (C-arm), 20 December 2025 (O-arm) and 18 June 2025 (CT); the three modalities were therefore not acquired on the same day. Parameters that could be verified from the retained records are summarised in Table 1. Parameters that were not retained are reported as not available rather than inferred. In the CT dataset, metal artefact reduction (iMAR) was part of the reconstruction. The CT dataset used for navigation was the exported sagittal multiplanar reconstruction (bone window, iMAR) with 2 mm slice thickness.

**Table 1:** Acquisition and reconstruction information available from the retained records

Parameter	C-arm (Ziehm Vision RFD 3D)	O-arm (Medtronic O-arm MVS O2)	CT (Siemens SOMATOM Drive)
Date of retained records	28 May 2025	20 December 2025	18 June 2025
Dataset / reconstruction	3D volume, 512 × 512 matrix, isotropic voxel 0.39 mm (pixel spacing and slice thickness 0.390625 mm), FOV 200 mm	Not available	Sagittal MPR, bone window, iMAR, slice thickness 2 mm, FOV 236 mm (source axial series: slice thickness 0.75 mm, kernel Br58, FOV 246 mm)
Tube voltage	Not recorded in DICOM header	50 kVp	100 kVp
Tube current / mAs	Not recorded in DICOM header	9 mA (2D header of retained image); mAs not available	Not available
Software version	7.07.6.1754	4.2.0	Not available
Navigation software version	Not retained	Not retained	Not retained
System-reported registration accuracy	No numerical value provided by NextAR	No numerical value provided by NextAR	No numerical value provided by NextAR

FOV, field of view; MPR, multiplanar reconstruction; iMAR, iterative metal artefact reduction. Values that could not be verified are reported as not available

### Navigation system and registration

Navigation was performed with the NextAR system, consisting of an infrared optical camera, a navigated pointer, a rigid patient reference marker (fiducial) and navigation software for three-dimensional visualisation. The fiducial was rigidly attached to the phantom. Image-to-patient registration was part of the mandatory NextAR workflow and was repeated for every acquisition; it was successful in all sessions. NextAR does not display a numerical registration-accuracy value: at the start of each registration the system confirms its accuracy check without reporting a value. Registration accuracy is, in any case, a property of the registration step and not an independent measurement of TRE at the target points. Pointer calibration was checked after each registration.

### Measurement procedure and definition of visually detectable pointer deviation

For each nail within the imaged volume, the tip of the navigated pointer was placed manually on the centre of the nail head. The pointer tip was localised visually, using the nail head (1 mm) and the underlying millimetre grid as reference. The operator then inspected the three-dimensional NextAR display. A deviation was judged absent when the pointer tip, placed centrally on the nail head, also appeared centrally on the nail head in the displayed image. Any visible offset of the displayed pointer tip from the centre of the nail head was recorded as a detectable deviation. NextAR does not provide a numerical deviation value; rounding or truncation therefore did not apply. No artificial or predefined registration error was introduced, and the pointer tip was not independently measured with an external reference system.

The primary endpoint, visually detectable pointer deviation (hereafter detectable deviation), was defined as a visible offset of the pointer tip from the centre of the nail head on the NextAR display. The threshold of about 1 mm is an approximate limit resulting from the size of the nail head and the millimetre grid. It was not established by calibration or by an independent measurement of the accuracy of the system. Smaller deviations could not be assessed. The endpoint is a visual, binary assessment by the operator and not an independently validated continuous TRE. It includes contributions from pointer calibration, manual pointer placement, optical tracking, image-to-patient registration and display resolution.

### Experimental unit and measurement structure

The experimental unit was one image acquisition including its image-to-patient registration. Ten acquisitions were performed per modality (30 in total), each followed by a new registration; the phantom and the fiducial were not repositioned, and target positions were reassessed in each new registration. Repeated acquisitions therefore reflect the variability of image acquisition and registration under fixed bench-top conditions, not the full variability of clinical practice (for example repositioning, different operators or patient anatomy). In each acquisition, every nail within the imaged volume was assessed once (28 nails with C-arm, 30 with O-arm and 40 with CT; see Results). Target observations within one acquisition share the same registration and were not treated as independent replicates. All measurements were performed by a single operator with extensive experience with all three imaging modalities; the operator was not blinded. Inter-operator variability was not assessed.

### Statistical analysis

The analysis was descriptive. For each modality and overall, the proportion of acquisitions with at least one detectable deviation was calculated with exact two-sided Clopper–Pearson 95% confidence intervals. Because all observations were below the detection threshold and the study was not designed as an equivalence study, no hypothesis tests or equivalence analyses were performed and no equivalence margin was prespecified. The confidence intervals refer to the acquisition level under the fixed bench-top conditions and do not reflect the complete variability of clinical practice.

## Results

Ten image acquisitions were performed for each modality (30 in total). Registration was successful in all sessions, and all planned measurements were completed without technical complications. NextAR provides no numerical registration-accuracy values; none are presented.

No detectable deviation was observed in any acquisition. The observed proportion was 0/10 for each of the three modalities (exact two-sided 95% CI 0–30.9%) and 0/30 overall (95% CI 0–11.6%; Table 2). The maximum fiducial-to-target distances evaluated were 143 mm (C-arm), 152 mm (O-arm) and 199 mm (CT). The ranges differed because the extent of the phantom captured in the imaged volume differed between modalities (FOV 200 mm for the C-arm, 236 mm for the sagittal CT reconstruction). Within the common range (up to 143 mm), no deviation was visible with any modality. The binary endpoint cannot quantify a distance effect below the detection threshold.

**Table 2:** Primary outcome by modality

Modality	Acquisitions (*n*)	Nails per acquisition*	Target observations	Max. distance (mm)	Acquisitions with detectable deviation	Exact 95% CI
C-arm	10	28	280	143	0/10	0–30.9%
O-arm	10	30	300	152	0/10	0–30.9%
CT	10	40	400	199	0/10	0–30.9%
All modalities	30	—	980	—	0/30	0–11.6%

The unit of analysis was the image acquisition with registration (denominator). Detectable deviation: visible offset of the pointer tip from the centre of the nail head on the NextAR display; the threshold of about 1 mm is an approximate limit given by the size of the nail head and the millimetre grid. Target observations within one acquisition are not independent and are given for description only. *Number of nails within the imaged volume, derived from the maximum distance: 14, 15 and 20 per row

## Discussion

In this controlled bench-top experiment with a flat two-dimensional phantom, no deviation of the pointer tip from the nail head was visible on the NextAR display after registration with C-arm, O-arm or CT imaging. The result is restricted to the tested phantom, acquisition conditions, operator and detection limit of the method.

The zero-event findings must not be read as proof of equivalence or absence of error. With 0/10 acquisitions per modality, the upper limit of the exact 95% confidence interval is 30.9%, so the data do not exclude a clinically relevant underlying rate of detectable deviations. Pooling all 30 acquisitions lowers the upper limit to 11.6% but does not change the descriptive nature of the study. The intervals refer to acquisitions under the fixed bench-top conditions used here and do not reflect the complete variability of clinical practice. Because every observation was below the detection threshold, the binary endpoint cannot show differences between modalities or distances.

The endpoint was deliberately based on what could be seen on the navigation display. The assessment was visual, performed by one unblinded operator, and limited by the size of the nail head and the millimetre grid. No independent reference system was used, and NextAR provides no numerical deviation. The study therefore cannot distinguish errors below about 1 mm and cannot establish submillimetre accuracy.

Previous studies have described differences in navigation accuracy between imaging modalities and have identified image quality, reconstruction and registration as possible contributors [7, 10, 13]. Neurosurgical literature also describes several sources of navigation error [20, 21]. The endpoint used here is not comparable with clinical screw-placement accuracy or with independently measured continuous TRE; the present results therefore neither confirm nor contradict those findings.

The literature suggests that TRE may increase with distance from the reference marker [15]. In the present experiment no deviation was visible over the evaluated distances. However, the endpoint cannot resolve changes below the detection threshold, and the maximum distance differed between modalities. Distances beyond the C-arm and O-arm coverage (143 and 152 mm) were available only for CT and must not be interpreted as a cross-modality comparison. The modalities also differed in voxel size and slice thickness (0.39 mm isotropic for the C-arm; 2 mm for the CT reconstruction used) and were acquired on different days; this is a further reason not to compare them directly.

The flat phantom allowed controlled testing of in-plane navigation display behaviour but does not reproduce the three-dimensional geometry of the spine. Depth, angular and trajectory errors were not assessed, and the findings cannot be extrapolated to pedicle screw accuracy or clinical navigation reliability.

### Limitations

First, the endpoint was a visual assessment limited to about 1 mm, so submillimetre deviations could not be quantified. Second, targets and pointer position were referenced to a millimetre grid and a 1-mm nail head, without an independent ground-truth measurement; the placement tolerance of the nails and the dimensional accuracy of the printed grid were not verified. Third, only 10 acquisitions were performed per modality with the phantom and fiducial fixed, so the precision of the modality-specific estimates is low and the confidence intervals do not reflect clinical variability. Fourth, one unblinded operator performed all measurements. Fifth, the phantom was two-dimensional. Sixth, the imaged distance ranges and the imaging parameters (voxel size, slice thickness, acquisition date) differed between modalities. Seventh, some acquisition parameters (e.g. mAs, tube voltage for some systems, O-arm 3D reconstruction parameters, navigation software version) were not retained and could not be reconstructed.

Future studies should use a three-dimensional anatomical or anthropomorphic phantom, ideally in cadaveric specimens, with an independent optical, mechanical or metrological reference system capable of measuring continuous three-dimensional TRE. More independent registrations and several operators would allow more precise estimates and an assessment of reproducibility.

## Conclusions

Under the controlled conditions of this bench-top experiment with a two-dimensional phantom, no deviation of the pointer tip from the target was visible on the NextAR display with C-arm, O-arm or CT imaging. The finding does not establish equivalence between modalities, submillimetre accuracy, absence of distance-related effects or clinical reliability. Verification with independent reference measurements in three-dimensional, cadaveric and clinical settings is required.

## Data Availability

The datasets generated and/or analyzed during the current study are available from the corresponding author on reasonable request.

## Abbreviations

2D: two-dimensional
3D: three-dimensional
CI: confidence interval
CT: computed tomography
DICOM: Digital Imaging and Communications in Medicine
FOV: field of view
iMAR: iterative metal artefact reduction
kVp: peak kilovoltage
mA: milliampere
mAs: milliampere-seconds
MPR: multiplanar reconstruction
TRE: target registration error
